# Insights into determinants influencing food security in the IGAD region of Eastern Africa

**DOI:** 10.3389/fnut.2025.1600096

**Published:** 2025-06-13

**Authors:** Paulino Omoj Omay, Titike Kassa Bahaga, Abdi Fidar

**Affiliations:** IGAD Climate Prediction and Application Centre (ICPAC), Nairobi, Kenya

**Keywords:** food security, food insecurity, determinants, IGAD region, East Africa

## Abstract

**Background:**

Food security in the Intergovernmental Authority on Development (IGAD) region of Eastern Africa is affected by a complex interplay of climatic and non-climatic factors. This study explores the major determinants of food security in the region, including extreme climate events (droughts and floods), land use, population growth, food production, market dynamics, and political and economic stability.

**Methods:**

The study employed a combination of descriptive and analytical approaches. Climatic data were derived from CHIRPS (1981–2023) to assess drought and flood patterns using the Standardized Precipitation Index (SPI). Non-climatic data, including population statistics, land availability, food production, trade data, and price trends, were sourced from FAOSTAT. The graphical data illustration, correlation analysis was conducted to examine the temporal patterns and relationships between food security determinants and outcomes such as cereal production, food prices, and undernourishment.

**Results:**

Findings reveal that droughts and extreme wet conditions significantly impact food security outcomes across IGAD countries. Rainfall and arable land showed the strongest positive correlation with cereal production. However, despite vast land resources, countries like Sudan and South Sudan have not fully utilized their agricultural potential. Population growth, unbalanced trade policies, and limited investment in agriculture contribute to high food prices and undernutrition. The correlation analysis indicates that economic stability and population dynamics are key influencers of food production and accessibility. Urban–rural population imbalances and policy gaps further exacerbate food insecurity risks.

**Conclusion:**

This study highlights the urgent need for a multi-sectoral and regionally coordinated approach to enhance food security in the IGAD region. Strategies should focus on climate-resilient agriculture, sustainable land management, inclusive economic policies, and food system innovations. Regional cooperation, targeted investments, and context-specific policy interventions are essential to reduce vulnerability and achieve sustainable food security.

## 1 Introduction

Food security remains one of the most urgent and complex development challenges globally (1), and this is particularly true for East Africa. While food security encompasses various dimensions including availability, access, utilization, and stability (2) it is increasingly influenced by a convergence of climatic, economic, political, and demographic stressors (3). In the East African region, these challenges manifest more acutely due to the region’s high dependence on rain-fed agriculture (4), recurrent climatic shocks (5), population pressure (6), and fragile governance systems (7). Population expansion not only directly affects food insecurity by increasing demand, but it also has an influence on food supply and access owing to land damage and resettlement arrangements (8). This, along with rising communities and land distribution laws, results in smaller farm holdings, making food production economically problematic (8).

Despite being endowed with fertile land, water resources, and a large agricultural workforce, East Africa continues to face chronic food insecurity (9). Approximately 80% of the population relies on subsistence agriculture (10), livestock, and fishing for their livelihoods, making them highly vulnerable to both climate variability and market disruptions (11). Over 50 million people across the Intergovernmental Authority on Development (IGAD) region are currently food insecure a number that continues to rise due to recurrent droughts, flooding, land degradation, displacement, conflict, and rising food prices (12).

The vulnerability of the food security sector is influenced by a variety of drivers and stressors (13), including climatic factors such as CO_2_ fertilization effects and precipitation changes (14), as well as non-climatic factors such as soil fertility, irrigation, demography, economic stability, and the sociopolitical environment (15). Other stresses and shocks include natural catastrophes, health troubles, and difficulties in sustaining a livelihood (16). All of these factors have a major influence on food insecurity risk (17). The problem is further exacerbated by the impacts of climate change, which has intensified the frequency and severity of extreme weather events (18). Changes in rainfall patterns, delayed onset and early cessation of the growing season (19), and extreme temperatures (20) have all led to significant reductions in crop yields and livestock productivity (21) as a clear evidence of strong relationship between food insecurity and climate change (22). Crop diversification and agricultural approaches can improve resilience and reduce the impact of shocks (23). Food production should guarantee that food is available to everyone, particularly underprivileged communities (24). These climatic pressures are interwoven with non-climatic stressors, including population growth (25), limited access to arable land (26), weak infrastructure (27), economic instability (28), and political unrest factors that collectively constrain food availability and accessibility across the East Africa region (29).

Notably, political instability and conflict remain critical yet underexplored determinants of food insecurity in East Africa (30). Studies have shown that in countries like Somalia, South Sudan, and parts of Uganda, food crises are often preceded by political upheaval rather than climatic events alone (31). Poor governance, corruption, and the weaponization of food aid have undermined humanitarian efforts, compounding the vulnerability of already at-risk populations (32).

Many studies in East Africa have assessed the impacts of food security on major crops (21), the challenges posed by climate change, economic crises, and conflicts (3), the failures of food systems (33), strategies to address the impacts of climate change (29), and issues related to hunger and food insecurity (12), as well as seed system interventions (34). These studies focused on food-secure households (35) but did not compare the determinants and root causes of food insecurity in the region. Therefore, this article focuses on the determinants of food security. While previous studies have examined the impacts of climate change, agricultural productivity, or specific food systems in isolation, there is a gap in understanding the combined effects of climatic and non-climatic factors on food insecurity across the IGAD region. Many existing analyses focus on national averages or single sectors, overlooking the complex, interlinked drivers that vary across space and time (36).

This study addresses that gap by adopting an integrated, multi-dimensional approach that combines climatic and non-climatic indicators such as spatial patterns of droughts and floods, population distribution, land use, agricultural and commodity production, food imports and prices, and political-economic stability. The goal is to uncover spatial disparities and identify the root causes and key determinants of food insecurity in the IGAD region.

The novelty of this research lies in its holistic analysis of food security drivers across scales, offering a spatially informed understanding that supports evidence-based decision-making in disaster risk reduction, climate adaptation, land use planning, and food systems strengthening. In doing so, the study also contributes to the achievement of Sustainable Development Goals (SDGs), particularly SDG 2 (Zero Hunger) and SDG 1 (No Poverty), while promoting equity, resilience, and policy accountability in East Africa. The remainder of the paper is organized as follows: Section 2 outlines the study area, data sources, and analytical methods. Section 3 presents the key results. Section 4 discusses the findings in light of existing literature and policy implications. Section 5 concludes with recommendations for future research and action.

## 2 Data and methods

This sub-section describes the data and methods used. The data discussed in 2.1, while methods are described in 2.2.

### 2.1 Climatic and non-climatic data

#### 2.1.1 Climatic datasets

The climatic datasets used in this study are Hazards Group (CHG) Infrared Precipitation with *in-situ* station version 2 (CHIRPS v2.0) datasets from the University of California at Santa Barbara (UCSB) (37). The CHIRPS v2.0 is a quasi-global, high-resolution (0.05°), daily, pentadal, and monthly precipitation dataset scaled spatially to eight IGAD countries. The monthly timescale 1981–2023 was selected to assess the annual and seasonal frequency and intensity of drought as one of the most climatic drivers affecting food security sectors. The detailed information on this dataset, algorithm, major input datasets, and processes is found in Funk et al. (37). The selection of these datasets was informed by important role played by changes and variability in patterns of rainfall onset, cessation in the study by Omay et al. (38), patterns of wet/dry days, and wet/dry spells on drought recurrent over the IGAD region (38).

#### 2.1.2 Non-climatic datasets

**Figure 1:**
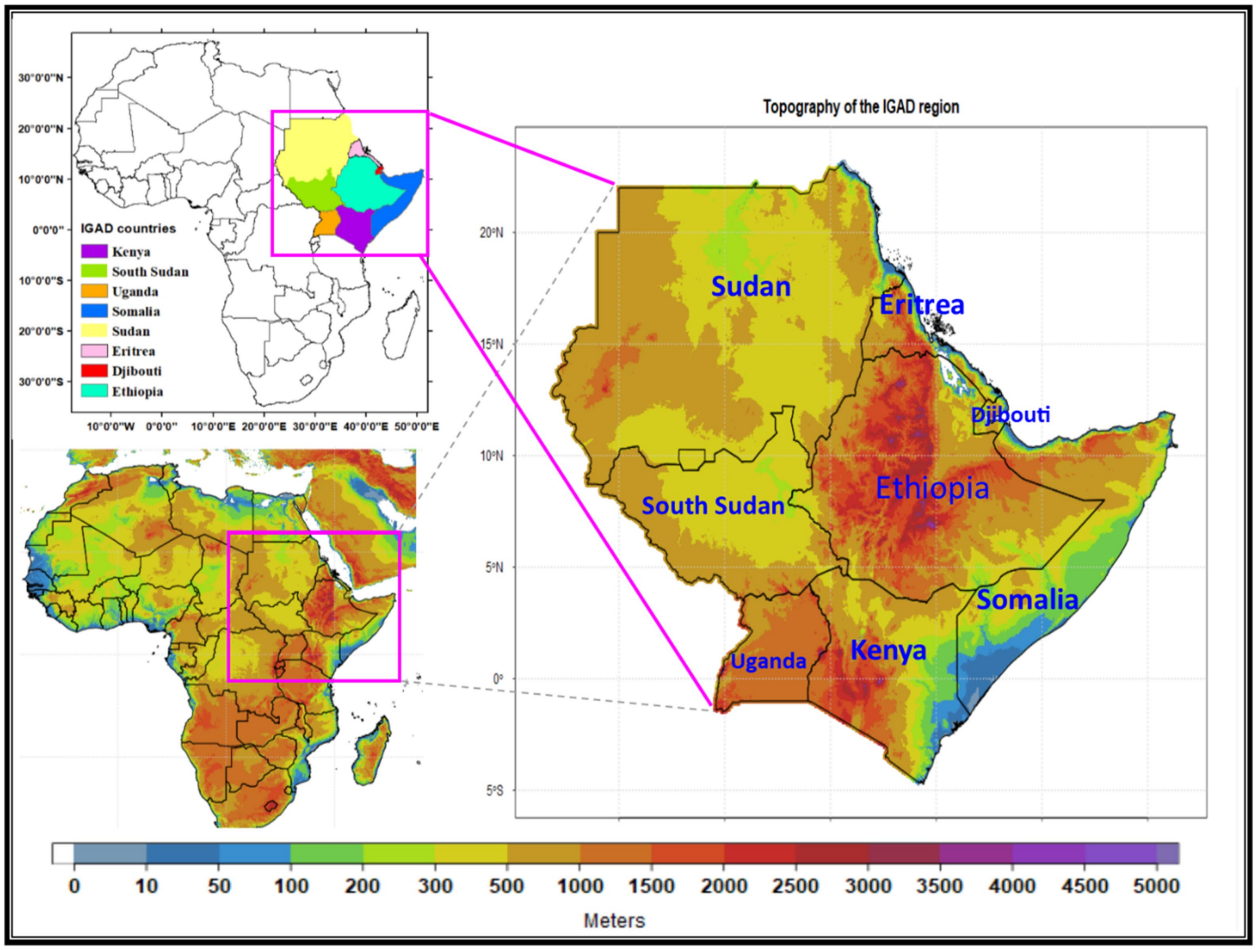
Shows the elevation (m) of the IGAD regions and countries, member states, region, and position in the African continent. The data source is the Digital Elevation Model (DEM) retrieved from the Shuttle Radar Topography Mission (SRTM).

Non-climatic indicators utilized in this study include population distributions, land factor, economic and political stability, food and commodity production, imports and exports, and food prices for eight IGAD countries. These datasets were obtained from the Agriculture Organization of the United Nations (FAO)’s FAOSTAT online portal FAOSTAT for the eight IGAD country levels. This analysis focuses on the key determinants and outcome indicators of food security in the IGAD region. It considers the various dimensions and components of food security, taking into account the level of analysis, data requirements, advancement in methods and techniques, and concepts in food security units of measurement (39). The indicators were chosen based on their direct relationship to food systems, production, and consumption patterns. The filtered and most relevant indicators, depending on the availability of data at the national level, were selected as composite indicators to measure the multidimensional concept and theoretical factors influencing food security (40). These indicators also describe the pathways and how they contribute to understanding the temporal patterns of food insecurity across the eight IGAD member states in the East Africa region (Figure 1).

The analysis of different climatic and non-climatic food security determinants datasets is driven by several key objectives and reasons. Droughts, floods, and shifts in population distribution directly impact agricultural productivity, food availability, and accessibility (41). Mapping the spatial patterns of these climatic events aids in developing early warning systems and resilience planning. Additionally, understanding land factors such as soil quality and land degradation is crucial for designing climate-smart agricultural strategies. Disruptions in food production and trade due to climate events or political instability can lead to economic shocks, particularly in agriculture-dependent economies. Food price volatility, reduced production, and disasters negatively impact dietary quality and health outcomes (42). Analyzing spatial patterns of climatic extremes can inform targeted mitigation strategies to address the challenges of climate change. Furthermore, a data-driven analysis of economic and political stability, in conjunction with climatic factors, helps ensure effective governance and resource allocation. Examining imports, exports, and commodity production reveals supply chain vulnerabilities and guides market interventions. Finally, analyzing population distribution alongside climatic data exposes disparities in exposure and adaptive capacity.

**Figure 2:**
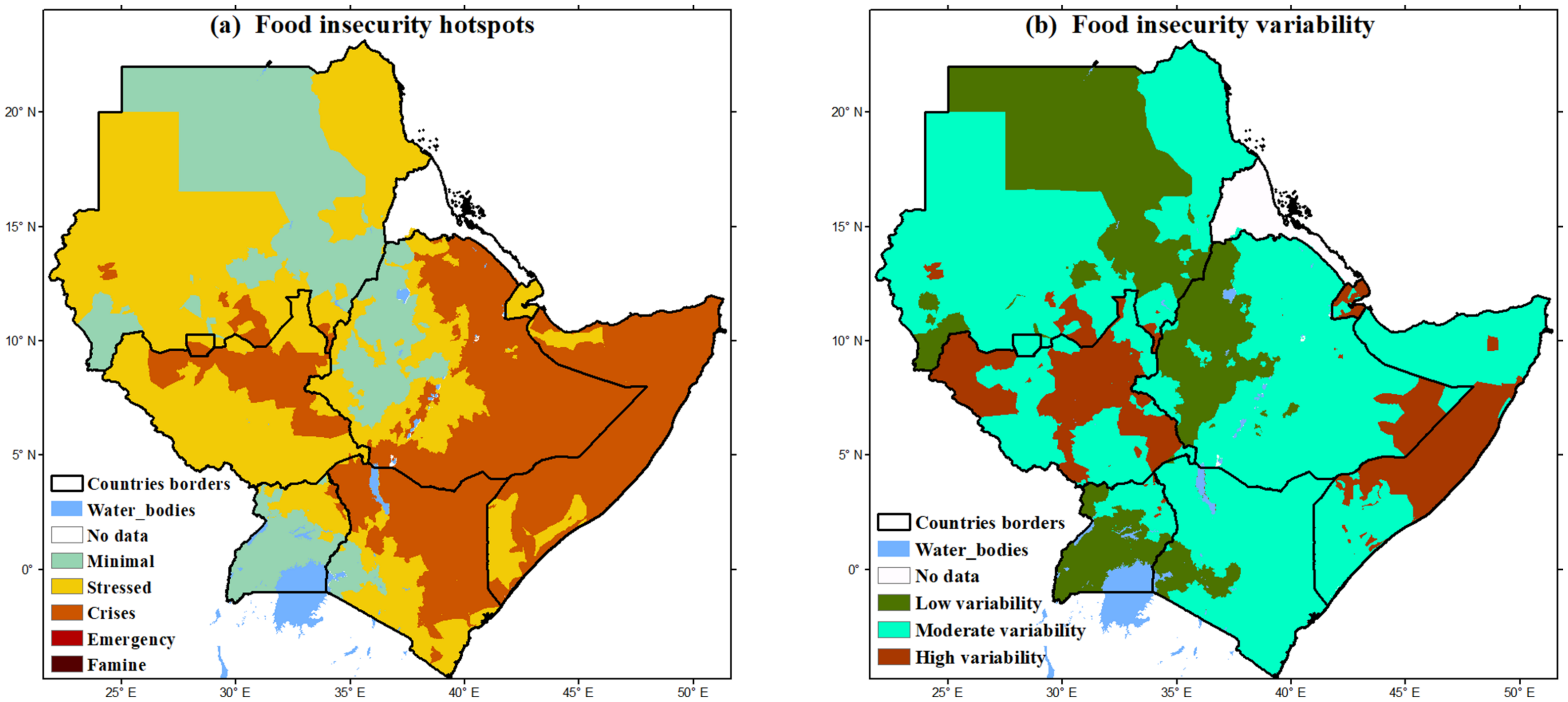
Spatial patterns of food insecurity hotspots (FIH) mean average and variability (2009 and 2019) over the IGAD region. Data source: FEWS NET Integrated Food Security Phase Classification (IPC). Where **(a)** are food insecurity hotspots, while **(b)** is food insecurity variability.

The conceptual frameworks (Figure 2) describe the cause- and-effect framework, which emphasizes the causal relationships between different food security determinants and how these determinants directly or indirectly impact food security. The determinants are divided into primary determinants, such as extreme climate events, population distribution, and land factors, and secondary determinants or feedback loops, which include economic and political stability. These factors influence food prices and the production, imports, and exports of commodities. Food production and prices are core elements impacting food security availability, access, utilization, and stability (43).

To confirm the status of vulnerability of IGAD region to food insecurity, the Figure 2 presents the baseline average of food insecurity hotspots (FIHs) across the IGAD region for the period 2009–2019. The spatial distribution of FIHs underscores the compounded influence of both climatic and non-climatic stressors in shaping food security outcomes across East Africa. Arid and semi-arid lands (ASALs) in Sudan, Eritrea, Djibouti, Kenya, Somalia, and southeastern Ethiopia consistently fall within the “minimal,” “stressed,” and “crisis” food insecurity phases, indicating chronic vulnerability and recurring hardship. In South Sudan, the expansion and persistence of FIHs are closely linked to ongoing conflict, political instability, and displacement, which have disrupted livelihoods, food systems, and humanitarian access. Meanwhile, in eastern and northeastern Kenya, southeastern Ethiopia, and most of Somalia, prolonged dry spells, high inter-seasonal rainfall variability, and recurrent droughts have triggered severe food crises. This observation aligns with findings by Fanzo et al. (1), which highlight the significant role of erratic rainfall patterns as a leading driver of food insecurity in the IGAD region. In contrast, relatively food-secure areas classified under the “minimal” category are concentrated in the wetter highlands of western Kenya and Ethiopia, and central and southern Uganda. These areas benefit from more reliable rainfall, higher rainfall totals, and extended wet seasons, which support agricultural productivity and buffer against seasonal food deficits. By visually contrasting these sub-regional disparities, Figure 2 illustrates why food insecurity in East Africa is not only more widespread but also more persistent compared to other parts of Africa. The combination of environmental fragility, demographic pressures, poor infrastructure, and political instability intensifies the food security challenge in this region, necessitating coordinated, context-specific interventions.

#### 2.1.3 Food security conceptual framework used in this study

**Figure 3:**
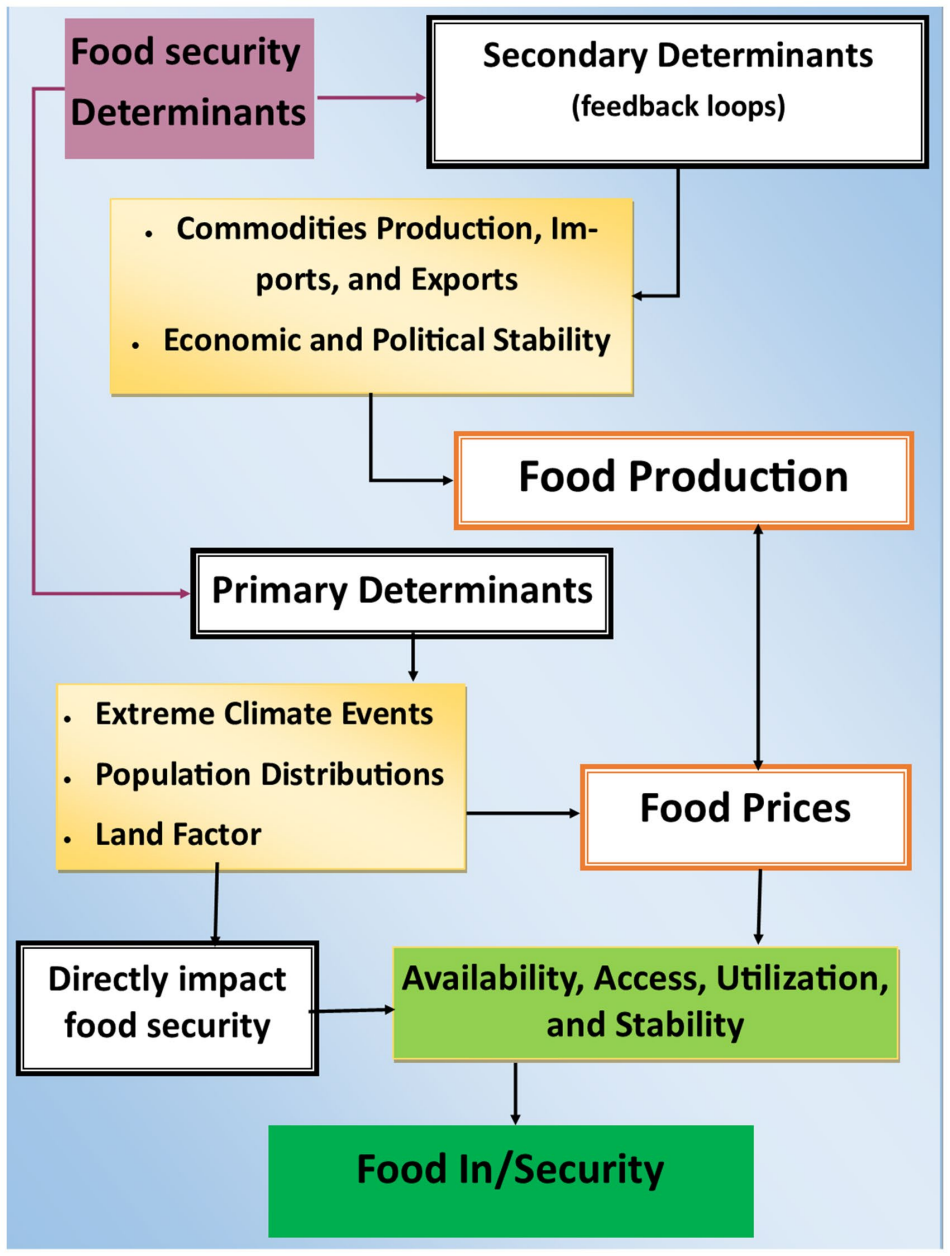
Conceptual frameworks illustrating the determinants influencing food security/food insecurity.

In the literature, there are different conceptual frameworks for food security. Most of the common frameworks are cause- and-effect framework, systems thinking framework, policy and governance framework, and temporal resilience framework. The food security conceptual framework used in this study is the cause- and-effect framework (Figure 3). The heart of the cause- and-effect framework lies in food security, conceptualized as the combined outcome of four interconnected components: availability, access, utilization, and stability. These pillars together define whether individuals and communities can consistently obtain and consume adequate food for a healthy and active life. Each of these elements is shaped by multiple upstream factors, some immediate and others more systemic. Understanding the causal chain that leads to food insecurity is crucial for developing targeted interventions.

The framework identifies three primary determinants that directly impact food security: extreme climate events, population distributions, and land factors. Extreme climate events, such as droughts, floods, and cyclones, can destroy crops, reduce agricultural yields, and disrupt food supply chains, directly undermining availability and stability. Population distributions affect demand and the geographic balance of food availability. Densely populated or urbanizing regions may face greater stress on food systems due to increased consumption needs and weakened local production. The land factors, including land availability, soil fertility, and land tenure systems, are central to food production. Degraded or poorly managed land constrains the amount and quality of food that can be grown, affecting both availability and access.

Secondary determinants function as indirect influences that often emerge from or intensify primary factors. Economic and political stability, for instance, plays a significant role in regulating food prices, market accessibility, and policy environments. Instability can trigger inflation, restrict trade, or limit investment in agriculture, all of which can make food unaffordable or unavailable. Similarly, food production is influenced by primary drivers like climate and land, but it also feeds into the broader economy by shaping commodities production, imports, and exports. Countries with limited production capacity may become dependent on food imports, making them vulnerable to global price shocks and supply chain disruptions.

### 2.2 Methods

#### 2.2.1 Standardized Precipitation Index

The Standardized Precipitation Index (SPI) measures precipitation anomalies across different time scales, including 1, 3, 6, and 12 months (44, 45). It is a valuable tool for evaluating drought and wetness conditions, both of which have direct implications for food security. By incorporating SPI into drought early warning systems, stakeholders can take proactive steps to address potential food insecurity. SPI aids in making informed decisions regarding crop selection, planting schedules, and irrigation requirements. Additionally, risk assessments based on SPI help prioritize regions for food aid during extreme climatic events. Data-driven insights from SPI also inform policies related to food security, water resource management, and climate adaptation. In this study, the SPI used to assess the patterns and intensity of drought in 2021–2022, widespread floods in 2023, rank severity of floods and drought years over each eight IGAD countries during last 42 years (1981–2023). The SPI computed based on gamma distribution, on multiple timescales (3, 4, and 12 months) to measure precipitation deficit, drought onset, duration, and intensity or surplus with respect to the long-term climate record (1991–2020). In addition, The SPI was calculated following the method proposed by Iwata et al. (45), which involves fitting a gamma distribution to the historical precipitation data and then transforming the cumulative probability to a standard normal distribution (mean = 0, standard deviation = 1). The Equation 1, which used to compute the SPI is presented mathematically as follows:

(1) $$\mathrm{SPI}=\frac{X-\bar{X}}{S}$$

where *X* is the precipitation for the period under consideration (1981–2023), $\bar{X}$ is the long-term mean precipitation, *S* is the standard deviation of the precipitation data.

#### 2.2.2 Graphical data illustration

Graphical data illustration involves the use of visual tools such as charts, maps, graphs, and plots to represent data patterns, trends, and relationships. It is particularly valuable in analyzing complex indicators. In this study, the graphical statistical methods used to understand the temporal patterns of droughts and flooding, trends in food security indicators (FIS), and food security related resources hypothesis testing. Histograms, box and scatter plots, pie charts, lines, and area bar charts are graphical statistical methods used to display trends and patterns of non-climatic factors linked to food security systems. These graphical statistical methods are powerful tools for data exploration, interpretation, and communication of results. The main purpose of displaying FIS graphically is to support a stated hypothesis of unused available resources in the IGAD region to tackle issues related to food security. The FIS displayed graphically are population growth, land and production systems, economic and political stability, food prices, and commodities being produced, exported, and imported. Visualizing these patterns and relationships, stakeholders in the IGAD region can design targeted interventions to address food insecurity, climate adaptation, and socio-economic challenges effectively.

#### 2.2.3 Pearson correlation

The Pearson correlation coefficient is used to compute the linkage between four key food security determinants, such as total rainfall, arable land, population, and economic stability, and three food security outcomes, which include cereal production, food producer prices, and the number of undernourished people in the IGAD region. The Pearson correlation coefficient is a statistical measure used to quantify the strength and direction of a linear relationship between food security determinants and food security outcomes. The food security determinants and outcomes for Ethiopia, Kenya, and Uganda were used as examples to provide more insights into the dynamic relationships between environmental, demographic, and economic factors influencing food security. The Pearson correlation coefficient is computed using the formula described in Equation 2.

(2) $$r_{\mathrm{xy}}=\frac{\frac{1}{n}\sum_{i=1}^{n}(x_{i}-\bar{x})(y_{i}-\bar{y})}{\sqrt{\frac{1}{n}\sum_{i=1}^{n}\{{\left(x_{i}-\bar{x}\right)}^{2}.\frac{1}{n}\sum_{i=1}^{n}{\left(y_{i}-\bar{y}\right)}^{2}\}}}$$

where $r_{\mathrm{xy}}$ is the Pearson correlation coefficient $\bar{x}$ and $\bar{y}$ are sample means of $x_{i}$ and $y_{i}$ . Where $r_{\mathrm{xy}}$ represent the correlation coefficient and *n* is the total number of observations of indicators. The value of $r_{\mathrm{xy}}$ range from −1 to +1 and is independent of the units of measurement. A value of $r_{\mathrm{xy}}$ near zero (0) shows less correlation between attributes; a value near +1 or −1 indicates a high (strong linkage) correlation food security determinants and food security outcomes.

## 3 Results and discussions

### 3.1 Hunger and food insecurity indicators

The IGAD region in Eastern Africa, comprising countries such as Djibouti, Eritrea, Ethiopia, Kenya, Somalia, South Sudan, Sudan, and Uganda (19), has faced significant challenges related to hunger and food insecurity over the past decades. Tables 1–3 provide a detailed analysis of hunger and food insecurity indicators, such as the number of people undernourished, which reflects the absolute number of individuals who do not have access to sufficient dietary energy (46); the prevalence of undernourishment (%), which represents the percentage of the population or chronic food insecure people, whose habitual food consumption patterns is insufficient to provide the dietary energy levels required to maintain a normal, active, and healthy life; and moderately and severely food insecure people, which combines both moderate and severe levels of food insecurity, capturing individuals who face uncertainties about their ability to obtain healthy food and have been forced to compromise on the type, quality and/or quantity of the food they consume daily (47). The analysis specifically highlights Ethiopia, Kenya, South Sudan, Sudan, and Uganda as examples.

**Table 1:** Hunger and food insecurity indicators: number of people undernourished in the IGAD region of Eastern Africa.

Years	Number of people undernourished				
	Ethiopia	Kenya	Somalia	Sudan	Uganda
2000–2002	32.2	10.3	6.4		4.6
2001–2003	31.3	11.1	6.6		4.3
2002–2004	31.1	11.8	6.9		4.2
2003–2005	29.4	11.2	7.1		4.3
2004–2006	28.7	10.2	7.4		4.7
2005–2007	28.4	9.7	7.6		5.3
2006–2008	28.7	10.3	7.8		6
2007–2009	28.2	10.6	8.1		6.3
2008–2010	24.5	9.8	8.3		6.1
2009–2011	21.6	8.8	8.4		5.9
2010–2012	18.7	8.2	8.3		6
2012–2014	17.8	8	8		6.6
2013–2015	16.8	8.1	7.8	3.5	7.5
2014–2016	15.5	8.4	7.8	3.5	9
2015–2017	14.8	9.4	8	3.7	11.3
2016–2018	14.9	10.3	8.3	4.2	13.3
2017–2019	16.8	11.2	8.4	4.5	14.2
2018–2020	20	11.1	8.3	4.8	14.2
2019–2021	23.7	11.7	8.3	4.9	14

**Table 2:** Hunger and food insecurity indicators: prevalence of undernourishment (%) in the IGAD region of Eastern Africa.

Years	Prevalence of undernourishment (%)				
	Ethiopia	Kenya	Somalia	Sudan	Uganda
2000–2002	46.7	32.3	70.6		18.4
2001–2003	44.1	33.7	70.6		16.9
2002–2004	42.5	35	70.5		16
2003–2005	39.1	32.1	70.4		15.9
2004–2006	37.1	28.4	70.4		16.9
2005–2007	35.6	26.1	70.4		18.5
2006–2008	35	27.1	70.4		20.1
2007–2009	33.5	27	70.5		20.7
2008–2010	28.2	24.3	70.9		19.4
2009–2011	24.2	21.3	70.1		18.4
2010–2012	20.3	19.2	68		18
2012–2014	18.9	18.3	64.2		19.3
2013–2015	17.3	18.1	60.4	9.7	21.3
2014–2016	15.6	18.3	58.7	9.3	24.7
2015–2017	14.5	20	58.2	9.7	30.2
2016–2018	14.1	21.5	57.7	10.6	34.2
2017–2019	15.5	22.8	56.6	11.1	35.5
2018–2020	18	22.2	54	11.5	34.1
2019–2021	20.7	23	51.9	11.3	32.5

**Table 3:** Hunger and food insecurity indicators: moderately and severely food insecure people in the IGAD region of Eastern Africa.

Years	Moderately and severely food insecure people				
	Ethiopia	Kenya	South Sudan	Sudan	Uganda
2014–2016_female	17	7.5		4.6	6.4
2014–2016_Male	16.2	6.4		4.5	6.4
2015–2017_Female	17.7	8.4		5.1	7.2
2015–2017_Male	17.9	7.1		5	7.2
2016–2018_Female	18	9.6	2.5	5.6	8
2016–2018_Male	19.6	8.2	2.4	5.5	8
2017–2019_Female	18	10.4	2.5	6.1	8.3
2017–2019_Male	19.9	8.9	2.3	6	8.3
2018–2020_Female	18.3	11.3	2.5	6.3	8.4
2018–2020_Male	19.8	9.6	2.3	6.2	8.5
2019–2021_Female	19.3	11.9	2.6	6.7	8.7
2019–2021_Male	20.1	10.1	2.4	6.6	8.8
2020–2022_Female	20.8	12.6	2.7	7	9.4
2020–2022_Male	21.2	11	2.5	6.9	9.3

The results show fluctuations in the number of undernourished individuals across the IGAD region from 2000 to 2021. In Ethiopia, the number of undernourished people decreased from 32.2 million in 2000–2002 to 14.8 million in 2015–2017, but there was an uptick to 23.7 million by 2019–2021. In Kenya, the undernourished population initially declined from 10.3 million in 2000–2002 to 8 million in 2012–2014, but rose again to 11.7 million by 2019–2021. In Somalia, the number remained relatively stable, hovering around 8 million, with slight increases in recent years. In Sudan, data collection began in 2013–2015, showing an increase from 3.5 million to 4.9 million by 2019–2021. In Uganda, the undernourished population grew from 4.6 million in 2000–2002 to 14 million by 2019–2021 (Table 1). These trends highlight that while some countries made progress in reducing undernourishment in the early 2000s, challenges persisted or re-emerged in subsequent years.

The prevalence of undernourishment (%) in the IGAD region (Table 2) shows that the prevalence decreased from 46.7% in 2000–2002 to 14.1% in 2016–2018, followed by an increase to 20.7% by 2019–2021 in Ethiopia. In Kenya, the percentage declined from 32.3% in 2000–2002 to 14.1% in 2016–2018 but rose to 20.7% by 2019–2021. In Somalia, consistently high rates were observed, with a slight decrease from 70.6% in 2000–2002 to 51.9% in 2019–2021. In Sudan, there was a gradual decrease from 70.6% in 2000–2002 to 51.9% in 2019–2021. In Uganda, the prevalence increased from 18.4% in 2000–2002 to 32.5% in 2019–2021. These percentages underscore the persistent and, in some cases, worsening food insecurity in the region.

The moderately and severely food insecure people in the IGAD region (Table 3) sheds light on the gender-disaggregated data of food insecurity from 2014 to 2022. In Ethiopia, both male and female populations experienced an increase in food insecurity, with females rising from 17% in 2014–2016 to 20.8% in 2020–2022, and males from 16.2 to 21.2% during the same periods. In Kenya, an upward trend is evident, with female food insecurity increasing from 7.5% in 2014–2016 to 12.6% in 2020–2022, and males from 6.4 to 11%. In South Sudan, data available from 2016–2018 shows relatively stable figures, with females at 2.5% and males at 2.4%, slightly decreasing to 2.7 and 2.5%, respectively, by 2020–2022. In Sudan, both genders experienced an increase, with females rising from 4.6% in 2014–2016 to 7% in 2020–2022, and males from 4.5 to 6.9%. In Uganda, food insecurity among females increased from 6.4% in 2014–2016 to 9.4% in 2020–2022, and among males from 6.4 to 9.3%. This gender-disaggregated data highlights that both males and females are increasingly affected by food insecurity, with females often experiencing slightly higher rates.

In summary, the IGAD region has faced numerous challenges and food insecurity determinants contributing to these trends. In the next sections, from 3.2 to 3.7, we analyse some of the common determinants believed to be the main reasons for the persistence of food insecurity in the IGAD region.

### 3.2 Spatial patterns of drought and floods

Analyzing the spatial patterns of extreme rainfall events is critical for understanding and addressing food security challenges in East Africa. The recurrent of droughts and floods are major causes of food insecurity in the East Africa region. Analyzing the spatial patterns of these events is essential to understanding their distribution, frequency, intensity, and impacts on agricultural systems, livelihoods, and food availability (48). Drought can turn chronic food insecurity into a famine. An example of this is the 1984 famine in Ethiopia where more than 1 million people starved to death (49, 50).

**Figure 4:**
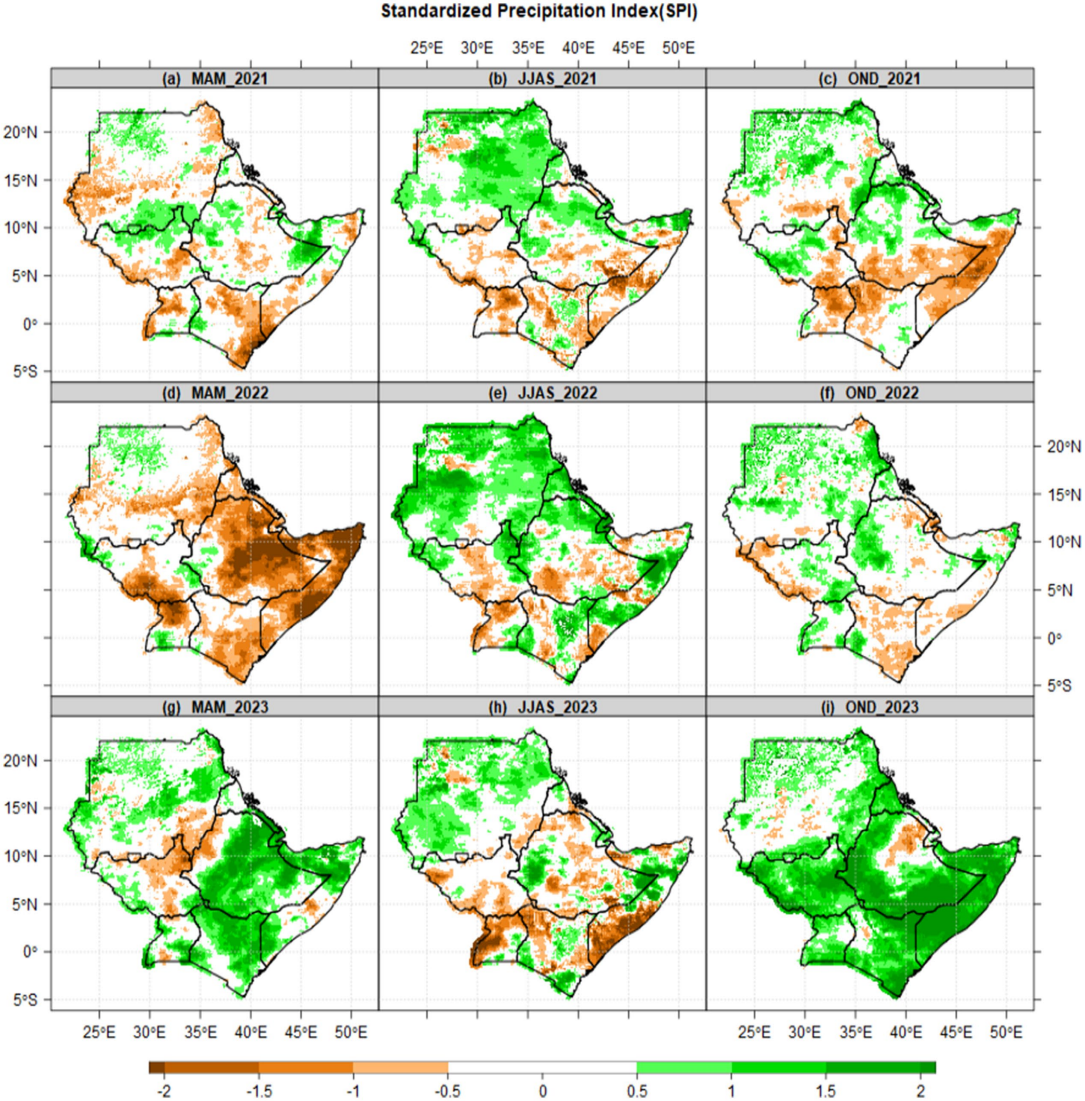
Spatial patterns of drought in 2021–2022 and floods in 2023 were analyzed using the Standardized Precipitation Index (SPI) relative to 1981–2010 baseline over the IGAD region of Eastern Africa. The legend indicates that the color green represents flooding, while the color brown represents drought. The SPI for 2021 is represented by raw one **(a–c)**, while raw two corresponds to 2022 **(d–f)**, and raw three pertains to 2023 **(g–i)**.

In recent years, countries such as Ethiopia, Sudan, Eritrea, and Kenya have all experienced severe food shortages due to drought. Due to the effects of El Niño and La Niña, East Africa has experienced consecutive droughts and floods over the past 3 years (2021–2023). The region, particularly the Horn of Africa, experienced mild to severe droughts in 2021–2022, and mild to severe floods in 2023 (Figure 4).

From 2021 to the end of 2022, East Africa experienced prolonged periods of below-average rainfall, resulting in failed March–May (MAM), June–September (JJAS), and October–December (OND) seasons. The drought affected several countries, including Ethiopia, Somalia, Kenya, South Sudan, and parts of Uganda (Figures 4a–f). In 2023, widespread and disastrous flooding occurred in northern and northeastern Kenya, as well as in most parts of Ethiopia during MAM (Figure 4g). The short rains in OND were extremely above normal, leading to widespread and disastrous flooding in most parts of the region (Figure 4i).

From the patterns of drought and floods in Figure 4, the arid and semi-arid regions (e.g., northern Kenya, southern Ethiopia, and parts of Somalia) are geographic areas prone to recurrent climate shocks. During drought periods, crop harvests are minimal, and there is a scarcity of water and pasture for animals. This leads to a decrease in the availability of food and an increase in food prices, exacerbating food insecurity and humanitarian challenges across the region. Pastoralist communities suffered heavy losses of livestock due to inadequate pasture and water resources. Understanding seasonal and long-term trends or patterns of drought and floods supports the development of adaptive measures, such as shifting agricultural zones or diversifying crops, resources allocation such as food aid, drought-resistant seeds, and irrigation systems can be deployed effectively.

### 3.3 Population distributions

**Figure 5:**
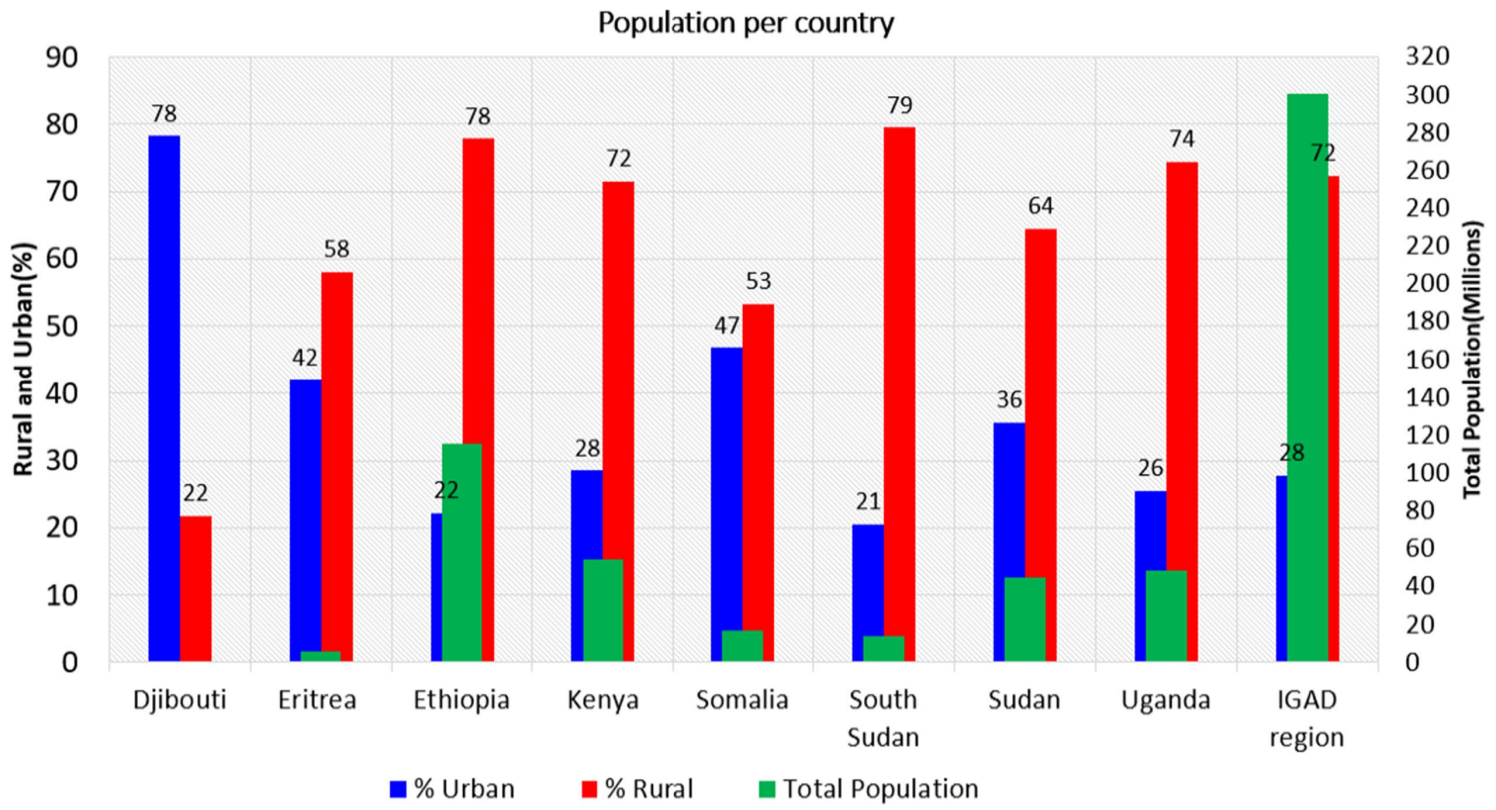
Total, urban, and rural population pattern in 2021 for eight IGAD member state. Data sources: processed raw data from FAOSTAT database.

The distribution of rural and urban populations in the IGAD region significantly impacts food security due to various interconnected socioeconomic, environmental, and infrastructural factors (51–54). The patterns of population distributions among eight IGAD member states are presented in Figure 5. Based on estimated 2021 population growth, the IGAD region’s population is approximately 300 million, with 217 million (72%) living in rural areas and 83 million (28%) residing in urban areas (Figure 5).

Ethiopia ranks as the most populous country in East Africa, with a total of 115 million people (78.2% rural and 28.8% urban), making it the second-largest population on the African continent (55). Following Ethiopia, Kenya has a population distribution of 28.5% urban and 71.5% rural, while Uganda has 25.6% urban and 74.4% rural. Sudan’s population distribution is 20.5% urban and 79.5% rural, Somalia’s is 46.7% urban and 53.3% rural, South Sudan’s is 20.5% urban and 79.5% rural, Eritrea’s is 42% urban and 58% rural, and Djibouti stands out with 78% urban and 21.8% rural. Djibouti is the only IGAD member state where the urban population outnumbers the rural population, which may be due to the surrounding environment, trade livelihoods, and better conditions for coping with extremely high temperatures in cities compared to rural areas.

Droughts, floods, and other climate shocks can devastate crops and lead to acute food insecurity (56). Limited access to markets due to poor infrastructure in rural areas and limited access to modern inputs (e.g., fertilizers, high-yield seeds) exacerbate the issue. Rapid population growth puts pressure on land and water resources, leading to land fragmentation, soil degradation, and reduced agricultural productivity (57). Urban populations are linked to food security through their reliance on rural areas for food supplies, which are vulnerable to climate shocks or transportation issues that can result in urban food shortages and price spikes (3). Food in urban areas is often more expensive due to transportation, storage, and market costs, disproportionately affecting poor urban households that spend a larger share of their income on food (58). Urbanization often leads to dietary shifts, with more reliance on processed and imported foods, increasing the risk of malnutrition and diet-related health issues (e.g., obesity, diabetes) if not balanced with fresh, nutritious foods (59). Many urban poor live in informal settlements with limited access to food markets, clean water, and sanitation, increasing their vulnerability to food insecurity and malnutrition.

The positive aspects of urban populations compared to rural populations include the following: urban areas create robust markets for agricultural products, incentivizing farmers to increase production and enhance efficiency. Cities often possess superior infrastructure, facilitating food storage, processing, and distribution, thereby improving food access. Furthermore, urbanization can generate non-agricultural employment opportunities, thereby augmenting household incomes and enhancing food affordability. Urban centers promote innovation, leading to advancements in food production, supply chain management, and food preservation techniques (60).

However, the patterns of urban population also present significant challenges to the food security sector. The high demand in urban areas, combined with limited local production, can drive up food prices, making it less affordable for low-income urban residents. Urban populations frequently rely heavily on imported food, rendering them vulnerable to fluctuations in global markets and disruptions in supply chains (61). Additionally, urban migration can result in labor shortages in rural areas, reducing agricultural productivity and jeopardizing local food production. The expansion of urban areas often encroaches on arable land, decreasing the land available for food production and straining water and energy resources. Conversely, rural populations serve as the backbone of agricultural production, supplying the majority of a region’s food. Rural areas typically have access to arable land for farming, which supports food security at both local and national levels (62). Rural communities often maintain indigenous farming practices that contribute to sustainable food production and biodiversity. Furthermore, rural populations engaged in pastoralism play a critical role in producing meat, milk, and other livestock-based food products.

Nevertheless, rural populations also face challenges to food security. Many rural farmers lack access to modern tools, inputs, and knowledge, leading to low yields and insufficient food production. Rural areas that are heavily dependent on rain-fed agriculture are particularly vulnerable to droughts, floods, and other climate impacts, which threaten food security. Additionally, rural households often experience high poverty rates and limited access to markets, constraining their ability to sell surplus food or purchase necessary supplies (29). The migration of rural youth to urban areas in search of better opportunities can further deplete the agricultural labor force, negatively impacting food production.

The interdependence between urban and rural populations is one of the factors influencing the status of food security in the IGAD region. Rural areas supply food to urban centers, while cities provide markets, thereby creating a symbiotic relationship that supports food security. Urban incomes and remittances from urban migrants can finance rural agricultural activities, thus improving productivity and resilience. Additionally, urban centers facilitate knowledge transfer and access to agricultural innovations, which benefit rural farmers. Conversely, urban areas often receive more investment and resources than rural regions, thereby widening inequalities and exacerbating food insecurity in rural communities. Moreover, inadequate infrastructure connecting rural and urban areas can lead to food losses during transportation and limit rural farmers’ access to urban markets. Furthermore, urban demand for water, energy, and land can divert resources away from rural areas, adversely impacting agricultural production and food availability. Urban and rural populations contribute to food security in complementary ways but also face unique challenges. Policies should prioritize rural–urban connectivity, equitable resource distribution, and targeted development to strengthen food security across the IGAD region.

### 3.4 Land factor

Land is essential for survival, wealth, power, and solutions for rapid population growth and to slow down the impacts of extreme rainfall events. The impacts of different land-use types—arable land, agricultural land, permanent crops land, meadows, and pasture land—on food security can be both positive and negative, influenced by environmental, socio-economic, and political factors. Arable land plays a crucial role in supporting crop production, which can increase the availability of staple crops (e.g., maize, sorghum, wheat), thereby enhancing the overall food supply. This type of land can also facilitate diverse cropping systems, which improve dietary diversity and provide economic opportunities, such as livelihoods for smallholder farmers, thus contributing to household food security. In addition, land dedicated to permanent crops supports the production of cash crops, including coffee, tea, and fruits (e.g., bananas, mangoes), which generate income for food purchases. Furthermore, permanent crop land contributes to foreign exchange earnings for governments, enabling them to finance food imports during periods of shortages.

Meadows and pastureland are essential for ensuring the availability of meat, milk, and other animal products, which improve protein intake. They also support pastoralist communities, which are integral to the food systems of arid and semi-arid regions. However, intensive cultivation of arable and agricultural land can lead to soil fertility depletion, reducing long-term productivity. Competition for arable land may result in disputes, negatively impacting food security. Additionally, expanding agricultural activities into marginal lands can result in deforestation and biodiversity loss, thereby diminishing essential ecosystem services such as pollination. The degradation of meadows and pastureland can lead to land degradation and desertification, particularly in semi-arid areas. Moreover, competition between pastoralists and farmers for resources can disrupt food systems.

**Figure 6:**
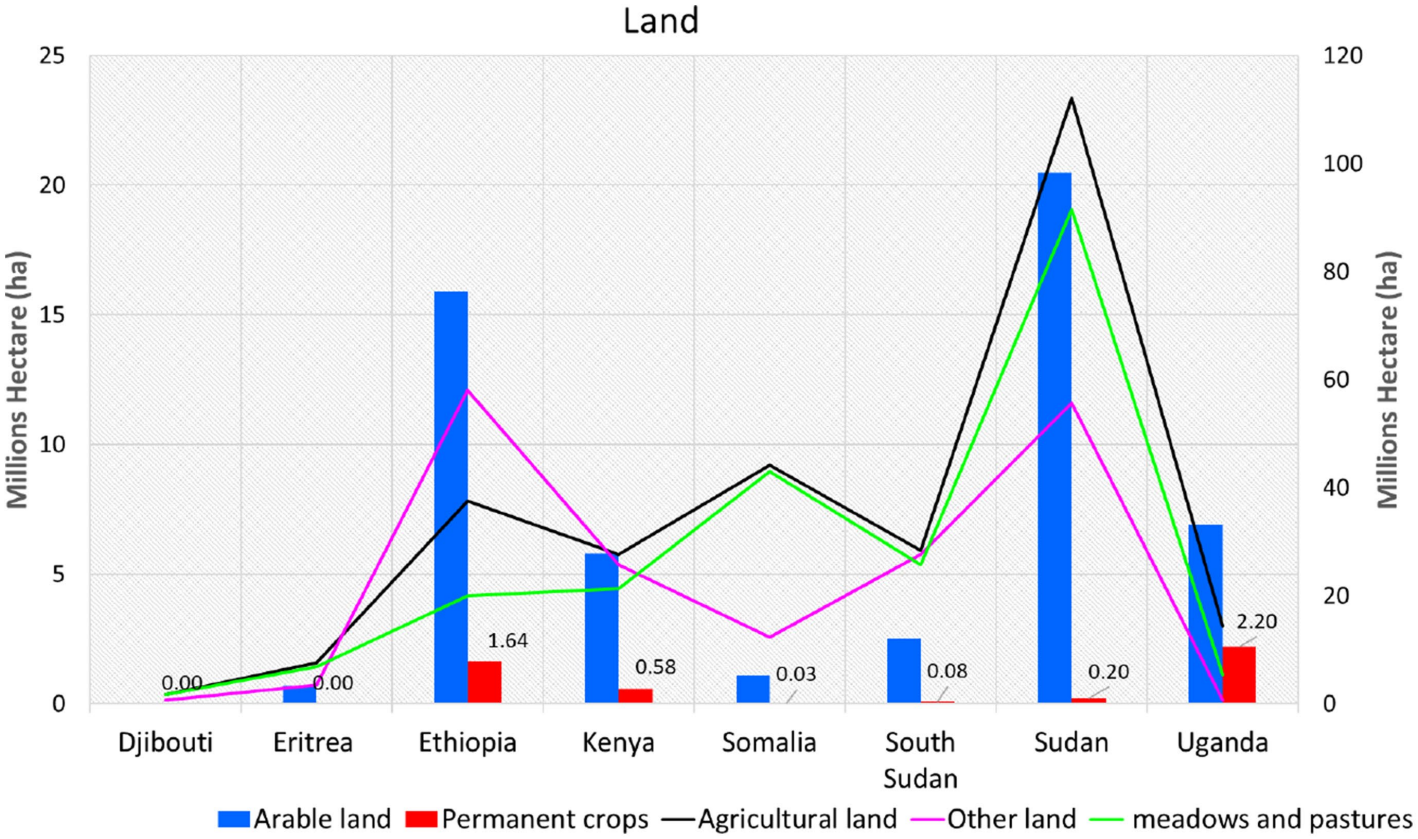
The patterns of agriculture, arable, permanent crops, meadows and pasture land in East Africa. Data sources: processed raw data from FAOSTAT database.

Figure 6 present the land opportunity offered by Sudan, Ethiopia, Uganda, Kenya and South Sudan to eliminate hunger and food insecurity in the IGAD region. The results show IGAD region has greater land opportunity for self-reliance food production and food export. Sudan is leading and followed by Ethiopia in arable land, agricultural land, permanent meadows and pastures land. Uganda is country in the region utilized his arable land to maximized permanent crops land, while Sudan and Ethiopia did not translate huge arable and agricultural land to permanent crops. Seems the unutilized land opportunities in the region, is the main source of food quantity imported, hunger and food insecurity. The rainfall season, which start in March to November is the main reason of highest land under permanent crops in Uganda.

The unimodal rainfall regime (June–September) posts less opportunity for Sudan to utilized huge agricultural and arable land into more production of food. If other option such as irrigation policies put in place, the land in Sudan and South Sudan could feed all population in East Africa. Therefore, sustainable land management practices, including conservation agriculture and rotational grazing, the implementation of diversified cropping systems, and the establishment of equitable land tenure systems to address land disputes, are essential for supporting sustainable food security in the IGAD region. The other factor is effective land government systems could be one of solution to improve food security. In addition, the public and privates’ investments could be one of best solutions to utilized the huge land and transformed it to permanents crops land (60). If very clear land policies, land tenure, property rights in place, the vulnerability to poverty, hunger, conflict and food insecurity will be an issue of past in the IGAD region of Eastern Africa.

### 3.5 Economic and political stability

Economic and political stability significantly influences food security outcomes in the IGAD region. Their impacts can be profound and multi-dimensional, affecting the availability, access, utilization, and stability of food systems.

Stability fosters conditions conducive to growth, equitable food access, and resilience, whereas instability exacerbates hunger, disrupts food systems, and heightens vulnerability. Stable economies promote investments in infrastructure, research, and modern agricultural techniques, thereby enhancing food production and availability. In the literature, numerous studies indicate that assessing the economic and political stability of a country necessitates a combination of quantitative and qualitative indicators that reflect governance, economic health, and resilience to shocks. Therefore, this study used the per capita food production variability and the values of food imports over total merchandise, the per capita food supply variability, economic and political stability and absence of violence/terrorism index to assess the effects of economic and political stability on food security status.

**Figure 7:**
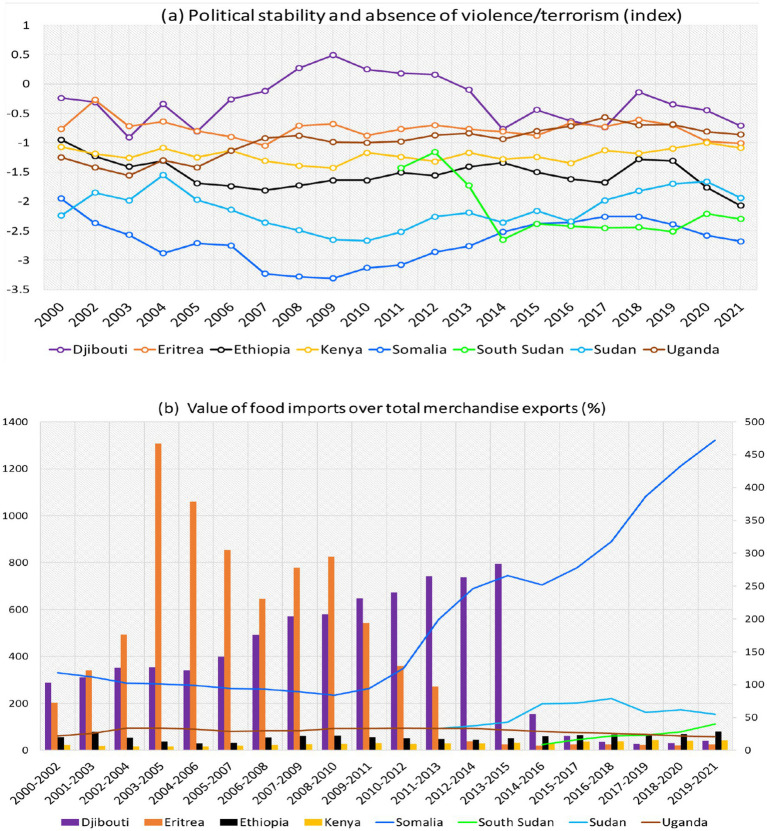
Times series political stability **(a)** and value of food imports **(b)** in total merchandise exports (%) (3-year average) over eight IGAD member states. Data sources: processed raw data from FAOSTAT database.

The results of political stability and values of food imports over total merchandise presented in Figure 7. The findings reveal that Djibouti is the most country stable politically (higher index), while Somalia with highest political instability over time. The conflict in South Sudan between 2014–2019 sent county to lowest political stability index in the region (Figure 7a). The economic and political stability have a large and direct influence on food supply, whether it is produced locally or imported from another location. During years of conflict and political instability, the food supply fluctuated greatly. For example, the value of food imports and per capita food supply variability is more stable in Kenya, Uganda, and Ethiopia. Djibouti and Somalia recorded highest value of food import between 2003–2013 (Figure 7b), with significant decrease from 2015 to date due to political stability in Djibouti and reduction of terrorism violence in Somalia. Food value import kept in increase over South Sudan since 2014 to present due to political unrest and conflicts.

The same results could be seen in Sudan with the highest variability in the value of food imports and per capita food supply due to country separation to two countries (Sudan and South Sudan) in 2011 (Figure 7b). Years of political turmoil, uncertainty, and anxiety generated by elections resulted in poor food production and fluctuation in per capita food supply, and increasing food costs. The combination of extreme rainfall events with political instability is a disaster for humanity due to the high values of food import, food supply from outside of country, and production variability, which reflect on food prices, food availability, and accessibility. Instability could introduce new behaviors such as biohazards or agroterrorism (deliberate introduction of animal or plant viruses into agricultural production systems with the purpose of causing economic harm and instilling public panic) studied in details by Djurle et al. (63). Political instability has not only hampered food security but have also bound almost all of the sustainable development agenda.

### 3.6 Food production

**Figure 8:**
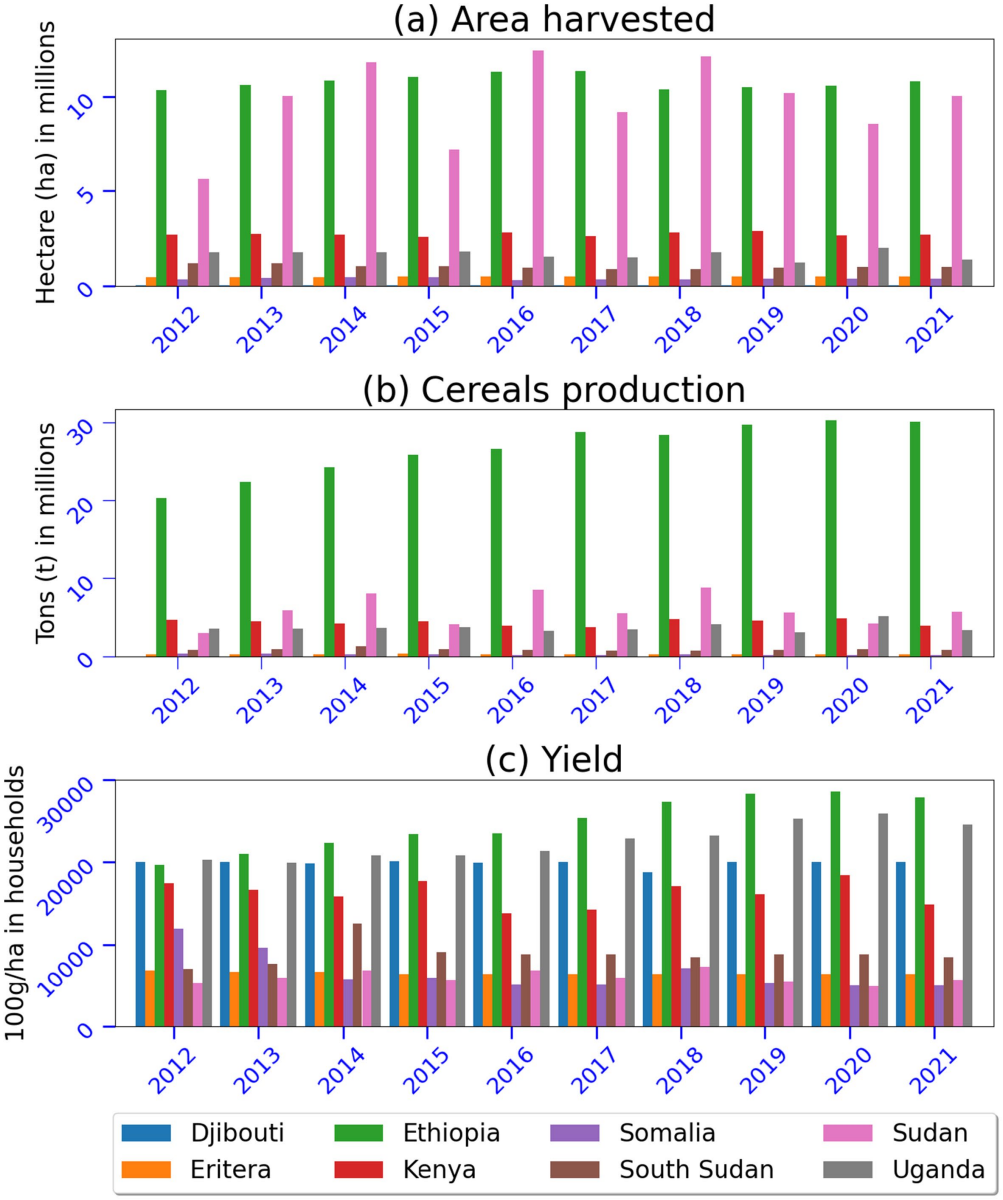
Histogram plots showing **(a)** harvested area, **(b)** cereals produced and **(c)** yields in eight IGAD member states. Data sources: processed raw data from FAOSTAT database.

The harvested area, cereal production, and yields play critical roles in shaping food security in the IGAD region. Figure 8 illustrates the harvested area; cereals produced and yields as example of food production indictors in eight IGAD member states. Sudan and Ethiopia are two nations in the region that have harvested over 10 million hectares of land. Sudan had the greatest annual fluctuation in harvested area (Figure 8a). Ethiopia leads the countries in cereal production and yields, which continue to rise year after year as a model for other countries (Figures 8b,c). With a higher harvested area, lower cereal output, and yields in Sudan compared to yields in Uganda, the results indicated that the intensity of heavy rainfall events had a greater influence in ASALs than in equatorial climate. Sudan led between 2014 to 2018, followed by Ethiopia; however, Ethiopia has recently taken the lead. Uganda leads in harvested area, yields, and cereal production, followed by Sudan.

Despite favorable climate conditions, arable and agricultural land potential, South Sudan has poor cereals output, yields, and harvested areas. Since 2012, it appears that the reliance on oil and political uncertainty have had an impact on yields and cereal output. High variability in rainfall events appears to be the major driver of high variability in harvested areas reported in Sudan, Ethiopia, Kenya, and Uganda. The patterns of harvested areas, cereal output, and yields compared to arable and agricultural land revealed the underlying cause of acute hunger, conflicts, and food insecurity in the IGAD region. In addition, unusually high rainfall in 2018–2020 and drought conditions in 2021 impacted food production and boosted/reduced areas harvested, cereal production, and yields. Another issue affecting agricultural productivity is the spread of diseases and the development of new crop pests as a result of changing rainfall patterns in the region (64).

The relationship between harvested area, cereal production, and yields significantly influences food security in the IGAD region, which predominantly relies on cereal crops as staple foods. Expanding the harvested area can result in increased total cereal output, thereby enhancing food availability and accessibility. Larger harvested areas may permit farmers to cultivate multiple crops, thereby enhancing dietary diversity and resilience to specific crop failures. Conversely, the expansion of agricultural land often occurs at the expense of forests and pastures, leading to soil degradation, water scarcity, and diminished ecosystem services that are critical for long-term food security. Furthermore, the increase in harvested area may exacerbate land-use conflicts, particularly between farmers and pastoralists. The cultivation of marginal lands may yield lower productivity, resulting in inefficiencies in food production. Higher cereal production enhances local supply, thereby reducing dependency on imports and bolstering food security. Increased cereal output can stabilize or lower market prices, making food more affordable for low-income households. Additionally, surpluses in cereal production may generate export income, thereby improving national economic stability and the capacity to invest in food security programs. However, overreliance on cereals renders food security highly susceptible to climate variability, such as droughts and floods. In situations of surplus where adequate storage or market access is lacking, cereals may go to waste, undermining the potential positive impacts on food security. Elevated production levels may depend on unsustainable farming practices, which can deplete soil fertility and water resources. Improvements in yields can enhance food production without necessitating additional land, thus conserving natural ecosystems and reducing land-use conflicts. Yield enhancements through climate-resilient crops and modern techniques may mitigate the adverse impacts of extreme weather conditions. Increased productivity can elevate farm incomes, enabling farmers to invest further in improving food security. Nonetheless, high yields often rely on chemical inputs, such as fertilizers and pesticides, which may be costly and environmentally damaging, thereby affecting long-term food security. Smallholder farmers, who frequently have limited access to inputs and technology, may struggle to achieve yield gains, exacerbating inequalities in food security. Intensified production practices may also lead to soil degradation, reduced biodiversity, and water pollution, thereby threatening sustainable food systems.

### 3.7 Commodities production, imports and exports

**Figure 9:**
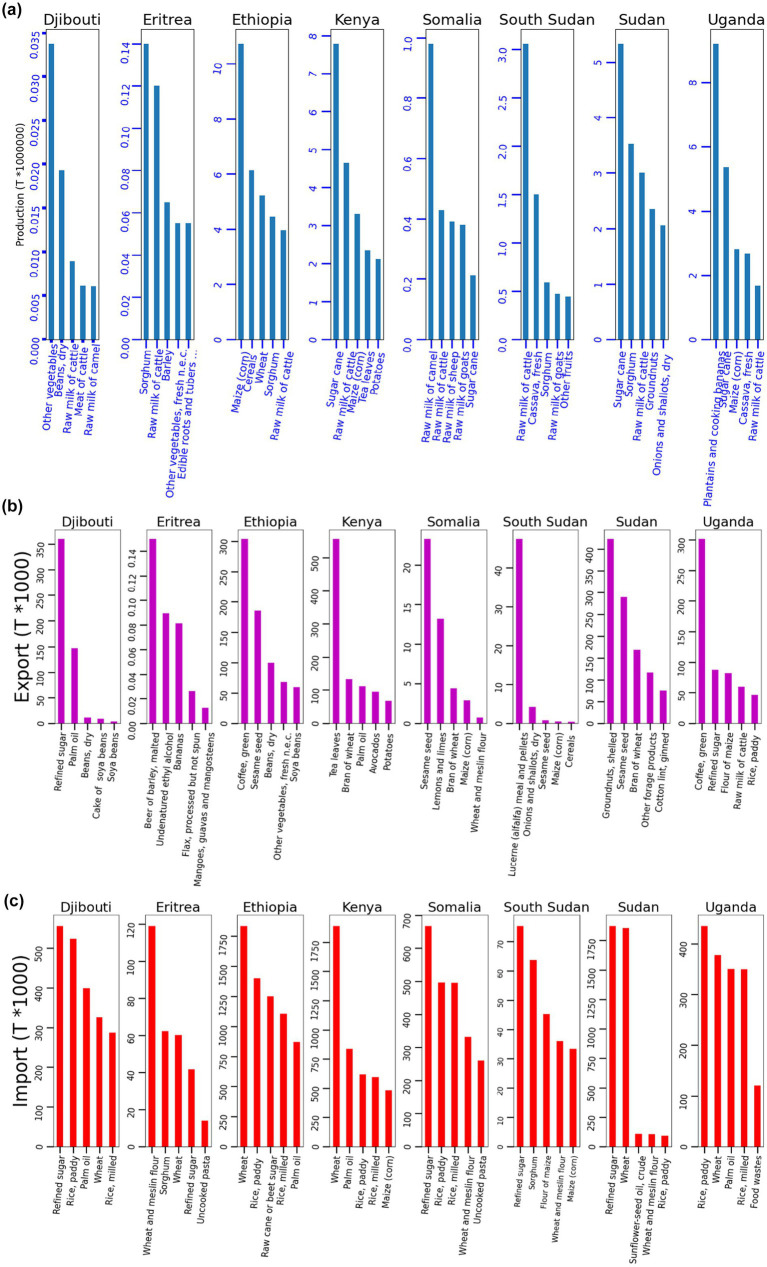
Histogram plots showing commodities production, import and export for eight IGAD member states in Eastern Africa (from left to right Eritrea, Djibouti, Ethiopia, Kenya, Somalia, South Sudan, Sudan, Uganda). Data sources: author processed raw data from FAOSTAT database. Where **(a)** represents commodities produced, **(b)** commodities imported, and **(c)** commodities exported.

The production, imports, and exports of food commodities play a significant role in shaping the main dimensions of food security (food availability, accessibility, utilization, and stability) and GDP at national and regional levels (65). The relationship between commodities production, imports, and exports is interconnected and complex, influenced by various factors. Figure 9 depicts the top five commodity production, imports, and exports by each country in the IGAD. Figure 9a illustrates the top commodities produced by IGAD countries. Djibouti’s top five commodities production includes vegetables, dried beans, raw milk from cattle, and cow meat. Eritrea’s top five commodities produced are sorghum, raw milk of cattle, barley, other vegetables, and edible roots and tubers with high starch. For Ethiopia, the top five commodities produced (the IGAD region’s most populous country) are maize (corn), cereals, wheat, sorghum, raw milk of cow, and barley. Kenya’s top five commodities production includes sugar cane, raw milk from cattle, maize (corn), tea leaves, and potatoes. In Somalia, the top commodities production includes raw camel milk, raw cattle milk, raw sheep milk, and raw goat milk. South Sudan’s top five commodities produced are raw milk of cattle, cassava, sorghum, and raw milk of goats. Sudan’s top exported commodities are sugar cane, sorghum, raw milk from cattle, groundnuts, onions and shallots, millet, and raw milk from goats. Uganda’s top five commodities production includes plantains and cooking bananas, sugar cane, maize (corn), cassava, and raw milk of cattle.

The positive impacts of domestic production of food commodities, such as cereals, fruits, vegetables, and livestock, directly increase the physical availability of food. High levels of agricultural production can reduce dependence on food imports, making countries more self-sufficient and resilient to global market disruptions. Increased production of food commodities can boost household income and purchasing power, improving access to food (66). It can also stimulate broader economic growth by creating jobs and improving rural livelihoods. When local production focuses on a variety of crops (including nutrient-rich crops like legumes, fruits, and vegetables), it can improve dietary diversity and nutritional outcomes. Greater diversity in local food production supports healthier diets and reduces reliance on staple crops alone.

The most imported commodities by countries in the IGAD are illustrated in Figure 9b. Djibouti’s top five imported commodities are refined sugar, rice, palm oil, wheat, and rice. Eritrea’s top five imported commodities are wheat and meslin flour, sorghum, wheat, refined sugar, and uncooked pasta. Ethiopia imports wheat, rice, raw cane, rice, and palm oil as its most significant commodities. Kenya imports wheat, palm oil, rice, maize (corn), refined sugar, and sorghum as its top import commodities. Somalia imports refined sugar, rice, wheat and meslin flour, uncooked pasta, and raw cane or beet sugar. South Sudan’s imported goods include refined sugar, sorghum, maize flour, wheat and meslin flour, and maize (corn), while Sudan’s top five imports are refined sugar, wheat, sunflower-seed oil, wheat and meslin flour, rice, and sorghum. The top five imported commodities in Uganda are rice, wheat, palm oil, rice, and food wastes.

The analysis of commodities imports shows that the majority of countries in the IGAD region depend on food imports, especially food staples like wheat, rice, and maize, that are not produced in sufficient quantities locally, helping to stabilize food availability. Imports can improve dietary diversity by providing access to food commodities that are not grown locally, such as fruits, vegetables, and animal products. This can help meet nutritional needs, particularly in regions with limited agricultural variety. During periods of poor harvests due to droughts, floods, or other climate-related events, imports can act as a buffer to maintain food availability and prevent severe food shortages (67). One of the negative aspects of heavy reliance on food imports is that it exposes countries to global price volatility resulting from geopolitical tensions and trade restrictions. The war between Russia and Ukraine is one recent example of geopolitical tensions that affected many countries in sub-Saharan Africa (5). Additionally, importing food requires foreign currency reserves, which can strain the balance of payments in low-income countries. If the cost of imports rises significantly, it can deplete foreign exchange reserves, leading to inflation and reduced government capacity to finance essential imports, including food.

The patterns of most exported commodities from the IGAD region to other countries are illustrated in Figure 9c. In Djibouti, refined sugar, palm oil, beans, soya bean cake, and soya beans are the top five exports. In Eritrea, beer of barley, undenatured ethyl alcohol, bananas, flax, processed but not spun, mangoes, guavas, and mangosteens are the top five goods exported. Whereas, coffee, sesame seed, beans, other vegetables, and soya beans are exported commodities from Ethiopia. Tea leaves, wheat bran, palm oil, avocados, potatoes, and sorghum are the top exported commodities from Kenya. Sesame seed, lemons and limes, wheat bran, maize (corn), wheat, and meslin flour are all exported commodities from Somalia. For South Sudan, the exported commodities include lucerne (alfalfa) meal and pellets, onions and shallots, sesame seed, maize (corn), and sesame seed oil. Groundnuts, sesame seed, wheat bran, various fodder products, cotton lint, pigeon peas, and groundnut oil are the most exported commodities in Sudan to other countries. The top five exported commodities to Uganda are coffee, refined sugar, flour of maize, raw milk of cattle, and rice.

Food export commodities, especially cash crops and high-value commodities (e.g., coffee, tea, and sugarcane) produced in Kenya, Sudan, and Uganda, are a major source of foreign exchange and national revenue. This income is normally reinvested in the agricultural sector or used to finance the importation of essential goods that are not produced locally. Strong export markets can also create jobs and boost rural economies, supporting the livelihoods of small-scale farmers and indirectly contributing to food security through increased household income. In some cases, food exports allow countries to take advantage of favorable global market conditions, generating revenue that supports broader food system investments, such as infrastructure, technology, and agricultural inputs (65). One of the negative impacts of food export in countries where a significant proportion of agricultural production is directed toward exports is that there is often less focus on producing food for local consumption. This can lead to food shortages and higher prices in domestic markets, even as the country exports large quantities of food. The shift to export-oriented agriculture can also deplete resources such as water and land, reducing the capacity for local food production. In addition, exporting food commodities can create domestic price volatility if international prices fluctuate sharply (68). Heavy reliance on a narrow range of export commodities can make countries vulnerable to international market fluctuations. For example, if demand or prices for a particular export crop (e.g., coffee or tea) decrease, it can result in a loss of income for farmers, reducing their ability to buy food and worsening food security. The patterns of production, imports, and exports show that the majority of countries in the region produced less food and imported the majority from outside. There is a clear imbalance in production, imports, and export quantities in the region. Investing in top commodities and locally consumed food is the best solution for sustainable food security instead of relying on imports.

The impacts of commodities production, imports, and exports on food security can be seen in increased food availability, boosted rural incomes, and enhanced dietary diversity when diverse crops are grown. Low production or a focus on cash crops can reduce food availability and worsen food security. Regarding food imports, they bridge food supply gaps and improve dietary diversity but increase vulnerability to global price shocks and trade imbalances. Meanwhile, food exports generate income and support economic growth, but overreliance on exports can create domestic food shortages and price volatility. The combined impacts of drought and floods, population growth, land policies, and economic and political stability on commodity production, imports, and exports create a feedback loop that exacerbates food insecurity and economic vulnerability in the Intergovernmental Authority on Development (IGAD) region. Droughts and floods, along with population growth, inadequate land policies, and instability, hinder agricultural production, increase import dependency, and diminish export competitiveness. Drought conditions reduce crop yields, devastate livestock populations, and deplete water resources, resulting in lower agricultural productivity. This leads to an increased reliance on food imports to address deficits caused by disrupted local production, loss of exportable surplus due to production shortfalls, and reduced competitiveness of agricultural exports as resources are diverted to meet domestic needs.

Population growth intensifies the demand for food and resources, placing significant strain on agricultural systems and other production sectors. As land and water resources are overexploited, long-term productivity is compromised. Higher import dependency arises to meet the rising food and commodity demands, and there is a shift toward importing processed or high-value goods due to urbanization and changing dietary patterns. Consequently, there is a decline in export volumes as commodities are redirected to satisfy increasing domestic consumption. Land policies significantly influence commodity production, imports, and exports. Weak or inequitable land tenure systems hinder investments in agriculture and sustainable practices, while inefficiencies in land use further reduce domestic food production, leading to increased reliance on imports. Poor land management can degrade export-oriented agricultural systems, negatively affecting the quality and quantity of exported commodities.

Economic and political stability also affect imports and exports. Conflicts and political instability disrupt production systems, damage infrastructure, and diminish labor availability. Economic challenges limit investments in modernizing production methods. Instability can escalate import costs due to disrupted trade routes, currency devaluation, and reduced purchasing power, while trade sanctions or conflict may restrict access to essential imports. Exports are likely to decline when conflicts disrupt supply chains or critical infrastructure, such as roads and ports. Furthermore, investor uncertainty in politically unstable regions hampers the growth of export-driven industries. Any country’s balanced commodity production, imports, and exports play a key role in the paths of food security, hunger, and food insecurity. Addressing the impacts of production, imports, and exports on food security requires policies that promote local food production, improve access to global markets, and mitigate the risks associated with dependence on food trade.

### 3.8 Food producer prices

**Figure 10:**
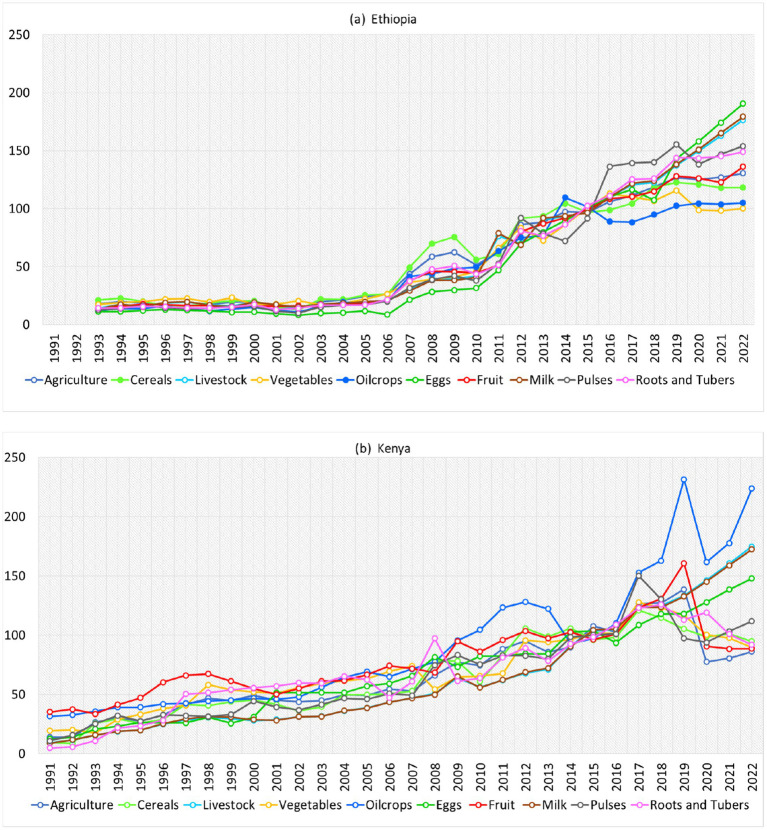
Times series showing surge in food prices in Kenya **(a)** and Ethiopia **(b)** for period 1991–2022. Data sources: author processed raw data from FAOSTAT database.

Food prices in the IGAD region are heavily influenced by interconnected factors such as droughts, floods, population growth, land policies, and economic and political stability, commodities production, imports and exports. Figure 10 shows the hike in food producer prices in Kenya and Ethiopia, serving as a sample of the results for all IGAD countries. The analysis only focuses on producers’ prices, as there is a prevalent and increasing trend of food producer price increase. This rise can be attributed to higher costs of inputs such as seeds, fertilizers, and labor. These increased costs are usually passed along the supply chain to wholesalers, distributors, and retailers, leading to an increase in food consumer prices and direct impacts on food security. The trends of food prices in recent years become alarming in most part of IGAD countries. Food prices determined by changes in production inputs directly or indirectly. Changes in producer and consumer food prices can directly affect households’ ability to access adequate, nutritious food (69).

In recent years, all counties in the IGAD region, recorded a surge and high variability in food prices compared to previous years. In Ethiopia (Figure 10a), the prices of food items have increased 100% (from 50-more than 100$) in last 4 years (2019–2022) compared to prices in 2010. Even some commodities such as eggs, milk and livestock have gone up three times compare to bassline of 2010. In Kenya (Figure 10b), oil crops, eggs, milk, livestock and cereals prices have gone up three times. Also, there is clear and significant impacts of government intervention measures in reduction of prices in Kenya. Generally, the food prices in the region considered as one of main driver of hunger and food insecurity. When food consumer prices increase, due to factors related to production inputs and other factors, the level of food access reduce, through reduction of purchase power at regional, national and household levels. Producer prices have an impact on consumer prices in the food supply chain. This impact can occur through different channels and pathways. One way is through direct cost pass-through mechanisms, where changes in producer prices directly affect retail prices. Another way is through indirect effects, which are influenced by market dynamics, competition, and government policies (70). The extent of this impact is dependent on factors like the structure of the supply chain, market conditions, consumer demand, and broader economic influences (71).

In summary, the fluctuations in food prices whether producer or consumer prices determine whether populations can access sufficient, safe, and nutritious food although measuring food security through composite indicators is still a promising area of research and scrutiny (72), however, the examining the relationships among droughts, floods, population growth, land policies, economic and political stability, commodity production, and international trade reveals significant determinants of food production, food prices, and food security. Our findings indicate that droughts diminish agricultural yields and livestock productivity, leading to supply shortages and price increases for staple crops such as maize, sorghum, and wheat. Conversely, floods devastate crops, disrupt transportation networks, and amplify post-harvest losses, thereby further inflating food prices. The resulting decrease in food availability exacerbates hunger and malnutrition.

Rapid population growth escalates demand for food, frequently outpacing domestic supply and driving up prices. Urbanization tends to shift consumption patterns toward more expensive processed and imported foods, contributing to higher average food costs. This rising demand exerts pressure on limited resources, deepening food insecurity, particularly among rural and low-income populations. The overexploitation of land and water resources undermines agricultural sustainability, posing additional threats to long-term food availability.

Inequitable land tenure systems and inadequate land management practices diminish agricultural productivity, leading to supply constraints and price surges. Conflicts over land use disrupt production and inflate food costs in the affected regions. Weak governance regarding land tenure undermines the resilience of smallholder farmers and their access to food. Further, displacement caused by land conflicts intensifies food insecurity among vulnerable populations.

Political instability and conflicts disrupt food supply chains, limit market accessibility, and result in speculative price inflation. Currency depreciation and inflation exacerbate the costs of imported foods, driving domestic prices higher. Displacement and restricted market access during conflicts further worsen food insecurity. Economic instability constrains investment in agriculture, impeding long-term food security initiatives. Low agricultural productivity heightens scarcity and escalates prices for local food commodities. Competition between export-oriented and domestic food production inflates costs, while insufficient production undermines food availability, particularly during crises. Moreover, export-focused agriculture can diminish access to food for local populations.

In conclusion, the IGAD region confronts interconnected challenges related to droughts, floods, population growth, land policies, economic and political stability, commodity production, and food pricing, which collectively perpetuate a cycle of heightened food insecurity. Addressing the determinants of food security requires investments in climate-resilient agriculture to stabilize production, equitable land reforms to enhance productivity and access, regional cooperation and stability to ensure efficient trade and food distribution, and the diversification of food systems to reduce reliance on imports and external markets.

### 3.9 Linkage between food security determinants and outcomes

This subsection examined the correlation between four key food security determinants such as total rainfall, arable land, population, and economic stability and multiple food security outcomes, including cereal production, food producer prices, and the number of undernourished people (72). The results provide insights into the dynamic relationships between environmental, demographic, and economic factors influencing food security in Ethiopia, Kenya, and Uganda (Table 1). Total rainfall showed a moderate correlation with cereal production, with values ranging from 0.29 in Ethiopia to 0.47 in Kenya. This underscores the critical role of rainfall in East Africa’s rain-fed agricultural systems, where seasonal variability affects crop yields (73). However, the correlation with food producer prices was weak, with values between 0.19 and 0.23, indicating that market forces, rather than direct rainfall fluctuations, have a stronger influence on pricing (74). Conversely, rainfall had a notable impact on undernourishment levels, with the highest correlation in Kenya (0.74). This suggests that rainfall shortages contribute to food insecurity through reduced agricultural output and increased vulnerability to droughts (75). Climate adaptation strategies, such as irrigation expansion and drought-resistant crops, are vital for mitigating these risks. Arable land demonstrated a strong positive correlation with cereal production (Ethiopia: 0.94, Kenya: 0.84, Uganda: 0.89), reinforcing its significance in determining food availability. Similar correlations were observed with food producer prices, ranging from 0.89 in Uganda to 0.94 in Kenya, suggesting that increased cultivation areas help stabilize prices by boosting supply (76). However, the relationship between arable land and undernourishment levels was weaker, particularly in Uganda (0.33). This implies that while land availability is necessary for food production, factors such as soil fertility, agricultural investment, and equitable access to land play crucial roles in addressing food security (77). Sustainable land management practices and policies promoting smallholder farmers’ access to land are essential for enhancing food security. Population growth showed a high correlation with cereal production (Ethiopia: 0.98, Kenya: 0.80, Uganda: 0.89), reflecting the increasing food demand driven by demographic expansion. The correlation with food producer prices was similarly strong, with values above 0.90 across all three countries, indicating that larger populations drive both production and market prices (78). However, its relationship with undernourishment was moderate (Kenya: 0.53, Uganda: 0.49), suggesting that while population growth increases food needs, its impact on food insecurity depends on food distribution systems, affordability, and government policies (69). Strategies such as enhancing agricultural productivity and improving food access mechanisms are crucial for ensuring that food production keeps pace with population growth. Economic stability exhibited the strongest correlation with cereal production, reaching 1.00 in Ethiopia, 0.84 in Kenya, and 0.93 in Uganda, highlighting the role of stable economies in agricultural investment and market efficiency (IMF, 2022). The correlation with food producer prices varied, being strongest in Kenya (0.92) and weakest in Ethiopia (0.44), suggesting that inflation and economic policies significantly influence price stability. Additionally, economic stability showed a moderate correlation with undernourishment (Kenya: 0.66, Ethiopia: 0.48), indicating that economic growth can enhance food security by improving household purchasing power and access to food (79). Policy interventions focused on financial inclusion, agricultural credit, and market infrastructure improvements are essential for ensuring long-term food security. This correlation reveals that economic stability, arable land, and population growth are the strongest determinants of food security outcomes in the IGAD region, while total rainfall remains a critical but less direct factor. The findings highlight the need for a multi-faceted approach to food security that integrates climate resilience, sustainable land management, economic policies, and agricultural innovations. Future research should focus on regional policy interventions tailored to specific country needs and assess the long-term impacts of climate change on food security dynamics (see Table 4).

**Table 4:** Linkage between food security determinants (total rainfall, arable land, population, and economic stability) and food security outcomes (cereal production, food producer prices, and number of people undernourished) in Ethiopia, Kenya, and Uganda.

Food security outcome	Food security determinants	Ethiopia	Kenya	Uganda
Correlation with cereal production	Total rainfall	0.29	0.47	0.37
	Arable land	0.94	0.84	0.89
	Population	0.98	0.80	0.89
	Economic stability	0.91	0.84	0.93
Correlation with food producer prices	Total rainfall	0.19	0.21	0.23
	Arable land	0.93	0.94	0.89
	Population	0.95	0.94	0.99
	Economic stability	0.44	0.92	0.58
Correlation with number of people undernourished	Total rainfall	0.54	0.74	0.71
	Arable land	0.49	0.55	0.33
	Population	0.7	0.53	0.49
	Economic stability	0.48	0.66	0.43

## 4 Discussions

In the literature, numerous food security indicators have been developed for use in research; however, there is no consensus among scientists or practitioners regarding the single ‘best’ food security indicator for measuring, analyzing, and monitoring food security (39, 40, 80). Therefore, this study analyzed climatic and non-climatic factors identified as primary determinants of food security and its outcomes (81). Climatic factors such as drought and floods, the trends of population growth, land and production systems, economic and political stability, food prices, and commodities being produced, exported, and imported significantly control food security status in the IGAD region by influencing food production, distribution, and access. These factors encompass a wide range of social, economic, political, and environmental variables (82). Extreme rainfall events such as droughts and floods, especially those driven by ENSO episodes, pose a significant risk to all components of food security everywhere in East Africa due to the strong association of these episodes with the occurrence of extreme wet and dry conditions (83). These occurrences could potentially affect agricultural production, devastate crops (23), and deplete water sources, resulting in food shortages and worsening levels of poverty (11), which were very clear during the devastating drought of 2021–2022, over Somalia, northeastern Kenya, and southeastern Ethiopia (84). The devastating impact of droughts and floods is projected to continue in the future, as indicated by the CMIP6 models (85). All countries in the IGAD region experienced a dramatic increase in rural and urban population, with Ethiopia leading with 115 million people (78.2% in rural areas and 28.8% in urban areas). The rapid increase in population in East Africa intensifies the struggle for scarce resources, including land, water, and pasture. These have direct and indirect implications for natural resources, land use, excessive grazing, decreased soil quality, decreased agricultural production, and food insecurity. By end of 2021, the East Africa estimated at 217,337,921 rural population (72.3%) and 83,255,573-urban population (27.7%), which can exacerbate food insecurity, and access to nutritious food to maintain a healthy and active life. This in agreement with research conducted by Hall et al. (86) suggests that the main driver of food insecurity and widespread undernourishment in Africa will be the projected rapid population growth. In the IGAD region, land constitutes a main factor in the processes of producing food for rapid population growth. If IGAD countries cooperate and work together to take advantage of the arable and agricultural land opportunities presented by Sudan, Ethiopia, Uganda, Kenya, and South Sudan, the issues related to hunger and food insecurity could become a thing of the past. Insufficient land availability, especially for small-scale farmers and marginalized groups, might impede agricultural efficiency and poverty (87). Disparities in land ownership, unstable land tenure arrangements, and land grabs intensify inequities in resource access, contributing to rural poverty and hunger (88). Implementing sustainable land management methods, such as soil conservation, agroforestry, and irrigation, alongside effective land use regulations and land tenure, is crucial for enhancing agricultural output and guaranteeing food security. On the other hand, governance, economic stability, and political stability are essential factors that facilitate food security by creating the required circumstances for investing in agriculture, accessing markets, increasing revenue, implementing social protection measures, developing infrastructure, preventing conflicts, and ensuring policy consistency (89). Food production is a function of population, land, and the political environment (88). Production determines communities’ availability, accessibility, diversity, nutritional value, resilience, livelihoods, and food quality. Strong food production systems increase the ability to withstand and recover from many shocks and crises, such as natural catastrophes, climate change, and economic downturns. Implementing a variety of cropping methods, utilizing robust crop types, and employing efficient water management measures may effectively reduce the negative effects of adverse events on food production and guarantee a consistent food supply, even in difficult situations (90). Also, food production influences the trends of commodities produced locally, imports, and exports and could have complex effects on food security at both national and global scales. Ensuring a stable supply of food and building resilience in the global food system require a careful balance between local production and international commerce. Enacting effective trade policies and creating policies for international cooperation and partnership. The cost of food has a direct impact on how affordable it is for consumers, especially for vulnerable communities that have limited means to buy it (91). Population growth, unconducive food production environments, and economic and political instability are the primary drivers of rising food prices in the IGAD region (92). Food price patterns put pressure on household finances, compelling families to dedicate a greater proportion of their income to buying food. Increased food costs diminish the capacity of low-income households to get a sufficient and nourishing diet, resulting in food insecurity and malnutrition. To effectively tackle food insecurity issues, comprehensive strategies are needed to address the root causes through policies and interventions aimed at improving people’s livelihoods, promoting sustainable agriculture, strengthening social safety nets, building resilience to shocks, and fostering inclusive and equitable development.

## 5 Conclusion

Drought and floods present substantial obstacles to food security sector, due to their effects on agricultural production, disruptions in the food supply chain, fragility of livelihoods, effects on nutrition, and health consequences. The fluctuation in climate patterns in IGAD region, exemplified by consecutive droughts in 2021–2022 and extensive floods in 2023 present difficulties for agricultural and food systems. Population expansion in the region exacerbates the burden on food production systems, pressure on agricultural land, water resources, food supply networks and related consequences on food demand, land use and competition, resource scarcity, urbanization, dietary changes, social and economic issues, policy, and governance. Various types of land in the region, effective utilization and implementing sustainable land management strategies such as crop diversification, soil conservation, water management, and agroforestry may improve agricultural output and help ensure food security. Producing food domestically guarantees a steady provision of food and minimizes reliance on imports. Strategies such as maintaining a balance between local production and imports and exports, adopting efficient trade regulations, and encouraging regional and international collaboration play a crucial role in achieving food security in the IGAD region. The food prices in the region are influenced by several variables such as weather conditions, agricultural yields, market speculation, trade policy, and geopolitical conflicts. To effectively tackle food security concerns, it is necessary to adopt a comprehensive approach that takes into account several elements including agricultural production, supporting smallholder farmers, access to agricultural inputs, food distribution systems, economic policies, environmental sustainability, and social equality.

## Data Availability

The raw data supporting the conclusions of this article will be made available by the authors, without undue reservation.
